# Electrophysiological Evaluation of Mirrored Hand Movements in Virtual Reality: A Proof of Concept for Virtual Mirror Therapy

**DOI:** 10.1007/s10548-026-01208-4

**Published:** 2026-04-28

**Authors:** Nuh Yilmaz, Mesut Canli, Tamer Demiralp

**Affiliations:** 1 Department of Physiology, Faculty of Medicine, Istanbul University, Istanbul, Turkey; 2 Hulusi Behçet Life Sciences Research Laboratory, Istanbul University, Istanbul, Turkey

**Keywords:** Reaching-Related Negativity, Center-Out Reaching Movement, EEG, Virtual Reality, Mirroring Visual Feedback, Neurorehabilitation

## Abstract

Observing the movement of the affected hand by virtually mirroring the healthy hand’s movement has been reported to accelerate motor rehabilitation. To investigate the underlying neurophysiological mechanisms, we recorded electroencephalograms (EEG) of fifteen healthy volunteers, who performed a center-out reaching task with their non-dominant hand while they saw the avatars of either the moving or the mirrored hand in an immersive virtual reality (VR) environment. The event-related potentials (ERP) and oscillations (ERO) time-locked to either the stimulus-cued initiation (sReach) or the finishing time point of the reaching trial (fReach) were compared between the direct and mirrored conditions. In sReach, a P3 wave was observed with larger amplitude on the motor areas controlling the mirrored hand (*p* = 0.01), while the amplitude of a negative potential shift preceding fReach time point, the reaching-related negativity (RRN), was also larger in the mirrored condition (*p* = 0.009). Finally, EROs in the theta, alpha, and beta frequency bands significantly differed between the two conditions (all *p* < 0.05) with scalp topographies pointing to either the activation of the motor areas controlling the mirrored hand or visuo-spatial attention system due to the incongruent visuomotor feedback in immersive VR. The results provide electrophysiological evidence for adaptive control processes underlying mirror therapy and extend classical models of movement-related EEG signals, offering a mechanistic bridge between virtual feedback and functional neurorehabilitation.

## Introduction

Mirror therapy (MT) exploits the visual illusion generated by observing the reflected movement of a limb placed along the mid-sagittal plane. This simple manipulation has been shown to enhance motor performance in the contralateral limb and has substantial therapeutic implications for individuals with hemiplegia or hemiparesis (Bahr et al. 2018; Mekbib et al. 2021). The clinical application of mirror visual feedback (MVF) was introduced by Ramachandran and Rogers-Ramachandran (1996) and has been characterized as a method for promoting neural plasticity and functional restoration by stimulating neural pathways associated with the affected arm by illusory visual feedback (Ramachandran and Altschuler 2009). The therapeutic utility of this approach has been further supported by clinical trials in stroke rehabilitation (Michielsen et al. 2011; Yavuzer et al. 2008), which suggest that mirrored feedback may engage latent motor pathways. A Cochrane review including 62 studies and 1,982 stroke patients demonstrated that MT can moderately improve motor function when used in combination with conventional rehabilitation techniques **(**Thieme et al. 2018). Despite these promising effects, the neural mechanisms by which mirrored visual feedback promotes functional reorganization remain only partially understood.

Recent theoretical frameworks in perception and action, particularly predictive coding, provide a mechanistic perspective that is well suited to explain the effects of MT (Friston et al. 2023; Kilner et al. 2007). In typical voluntary actions, the sensory consequences of movement generate a reafference that matches the efference copy—an internal forward model of the motor command. When sensory feedback is experimentally altered, as in mirror therapy or virtual mirroring, the visual reafference becomes incongruent with the efference copy and the proprioceptive reafference. Such multisensory mismatches are predicted to evoke large prediction-error responses in motor systems that normally should control the mirrored hand, generating updates in internal models and thereby modulating motor system excitability (Bays and Wolpert 2007; Kilner et al. 2007; Von Helmholtz and Nagel 2015). Characterizing the electrophysiological correlates of these mismatches may therefore shed light on how MT induces sensorimotor reorganization.

Virtual reality (VR) provides an advantageous platform for investigating such mechanisms. VR enables precise manipulation of visuo-motor contingencies and creates ecologically valid yet highly controlled environments for studying motor control. In particular, center-out reaching tasks—commonly used to probe continuous sensorimotor transformations—are especially relevant for studying the mechanisms of MT, because they strongly rely on the efference–reafference loop (Garipelli et al. 2021). Additionally, leveraging immersive VR allows for exact temporal alignment of visual and motor events with neurophysiological measurements, making it possible to isolate neural signatures associated with mirrored versus veridical visual feedback.

Electroencephalogram (EEG)-derived metrics such as event-related potentials (ERPs) and event-related oscillations (EROs) offer valuable tools for probing sensorimotor processes. One of the ERP components, the P3 (or P300) wave is of particular interest as a robust index of cognitive workload and attentional resource allocation. As established by the foundational work of Kok (2001), the P3 amplitude serves as a sensitive measure of the ‘processing capacity’ recruited during the evaluation of task-relevant stimuli. In the context of motor control, this framework suggests that as the complexity of stimulus-response mapping increases—such as during the integration of incongruent visual feedback—the brain must allocate greater neural resources, a process reflected in the modulation of the P3 component. On the other side, classic motor-related ERP components including the Bereitschaftspotential (BP) and contingent negative variation (CNV) reflect preparatory activity prior to movement onset (Deecke 2007; Shibasaki and Hallett 2006; Walter et al. 1964). Additionally, oscillatory modulations—such as event-related desynchronization (ERD) and synchronization (ERS) in various frequency bands—have been linked to self-paced and cued movements (Borra et al. 2023; Cavanagh and Frank 2014; Chatrian et al. 1959; Chung et al. 2017; Kormendi et al. 2021; Neuper and Pfurtscheller 2001). However, most existing work has focused on preparatory or post-movement phases of an instantaneous movement, leaving the EEG correlates of continuous reaching movements—especially under conditions of altered visual feedback—relatively under-characterized. The neural signatures associated with movement initiation and target acquisition during reaching movements in VR environment have been investigated to a limited extent (Garipelli et al. 2021).

In the present study, we recorded EEG during a center-out reaching task, performed in immersive VR under two conditions: (i) observation of a veridical hand avatar and (ii) observation of a mirrored hand avatar across the mid-sagittal plane. By time-locking EEG signals to both the start and the end of each reach, we sought to dissociate neural processes associated with movement initiation and target attainment. Using these features, we tested the following hypotheses:

1. Compared with veridical feedback, mirrored visual feedback will elicit distinct EEG signatures at both movement onset and movement completion, reflecting altered visuo-motor contingencies.

2. These differences may arise from two complementary processes:

a. recruitment of neural resources involved in controlling the visually mirrored hand avatar by the prediction-error signal generated by the illusory visual feedback, and.

b. increased visuo-spatial attention required to resolve discrepancies between visual reafference and the efference copy in the mirrored condition.

## Methods

This article explores the potential of VR-based mid-sagittal plane mirroring in promoting motor rehabilitation by analyzing ERP and ERO measurements during VR-based exercises to shed light on the neural foundations of such approach. In addition, it may offer insights into the development of individualized therapy protocols. By evaluating the findings obtained from healthy participants, this research aims to establish a normative neurophysiological baseline, which may help contextualize the theoretical mechanisms underlying the application of similar exercise protocols in clinical populations.

###  Participants

The sample size was selected based on previous EEG studies using comparable virtual reaching tasks (Garipelli et al. 2021). Given the literature indicating that biological gender (O’Boyle and Hoff 1987) and hand preference (Niebauer et al. 2002) may influence the effect of mirroring, to ensure a high signal-to-noise ratio and minimize physiological variance in this proof-of-concept study, 15 right-handed healthy male participants who had normal, or corrected-to-normal vision were included in the study. This control was implemented to avoid the potential modulatory effects of ovarian hormone fluctuations on motor cortex excitability and neuroplasticity (de Souza et al. 2022; Faustino et al. 2022; Smith et al. 1999). To determine hand dominance and ensure participants were not ambidextrous, validated for Turkish population version (Atasavun Uysal et al. 2019) of the Edinburgh Handedness Inventory (Oldfield 1971) was used before EEG recordings. This study was approved by the Clinical Research Ethics Committee of Istanbul University Istanbul Faculty of Medicine (2022/284).

### Virtual Environment Design

The virtual environment (VE) was created using Unity Engine (v2023.2.17f) and presented to the participants through the Meta Quest 2 headset. The headset provided 1832 × 1920 pixels resolution per eye and features 4 infrared cameras for position tracking with six degrees of freedom that are connected via Meta Quest Link software to a laptop equipped with an Intel Core i7-12700 H processor, 32 GB RAM, and an NVIDIA GeForce RTX 3050 Ti graphics card. When the experiment began, five white target spheres were placed on a circle with a 5 cm radius centered on a black sphere (starting point) at angles of 30°, 60°, 90°, 120°, and 150°.

### Experimental Design

The participants had to move their index finger from the starting point to one of these target objects and return back, and the elapsed time in milliseconds to reach the target was displayed after each execution. To minimize order effects and prevent any learning effects, the KuTools for Microsoft Excel was used. This allowed for the random selection of blocks and trials, with 1000, 1500, or 2000 ms durations between trials (inter-trial interval, ITI). The experiment consisted of 14 blocks each consisting of 10 trials. The first 2 blocks were training for the direct (DIR) and mirrored (MIR) conditions, while the remaining 12 blocks consisted of 6 DIR and 6 MIR blocks in a random order.

#### Direct (DIR) Condition

Participants performed the task with their non-dominant hand while the avatar of their non-dominant hand was displayed in VE.

#### Mirrored (MIR) Condition

Participants performed the task with their non-dominant hand while the avatar of their dominant hand was displayed in VE.

At the start of the experiment, participants who sat in front of a real table that has same measurements of virtual one in a stable position and adjusted the virtual table to a comfortable position to perform center-out reaching movements. The participants displayed the movements of their hand in a stable position. Participants waited at the starting point with their index finger until one of the five white objects’ color (target) turned blue. Then, participants had to initiate the reaching movement towards the target. When the participants reached the target, the target’s color turned to green from blue for 1 s. After 1 s, target’s color turned white that prompted participants to return to the starting point. For each trial, the time to reach the target object (RT) was calculated in milliseconds as the duration for the target object’s color blue to green.

### Trigger Apparatus

Because modern computers lack parallel ports, a system that enables low-latency data transfer, trigger marks related to stimuli presented in the VE in the present study were sent to the EEG recording device via custom-developed hardware, which is a Teensy 4.1 device compatible with the Arduino system that enables sending immediate signals through a USB port with below 1 ms delay.

### EEG Recording and Preprocessing

The EEG signal was recorded using the BrainAmp – Standard amplifier system (*BrainAmp Standart*,* Brain Products GmbH*, 2019) with 30 Ag-AgCl electrodes placed according to the extended 10–20 system referenced to electrodes placed on both earlobes. Electro-oculogram (EOG) signals were recorded between two electrodes placed on the upper and lateral orbital areas of one eye to correct eye movement artifacts. The data was filtered between 0.01 and 500 Hz and digitized at 1000 Hz. Electrode impedances were kept below 20 kΩ and were periodically checked throughout the experiment. The VR headset was securely placed over the electrode cap.

Pilot testing prior to the main experiment confirmed that the placement of the VR headset and its elastic band provided additional stabilization to the EEG cap, minimizing mechanical artifacts without inducing electrode bridging. Still, we eliminated EEG artifacts as strictly as possible with a preprocessing pipeline according to the procedures described by Luck (Luck 2022), using customized EEGLAB (Delorme and Makeig 2004) and ERPLAB (Lopez-Calderon and Luck 2014) scripts running under MATLAB. Eye-related artifacts were corrected with ICA. A bandpass filter of 0.1–45 Hz was applied to attenuate slow drifts (e.g., from headset pressure), high-frequency electromyographic (EMG) artifacts related to head or facial muscle activity and possible line noises. Continuous data were scanned automatically using a sliding window of 200 ms with 50 ms steps. Time windows were marked if the peak-to-peak voltage exceeded 200 µV without rejection and visually checked to identify whether real artifacts. Lastly, data was visually inspected to identify artifacts that were not marked by automatic algorithms and artifactual time windows were rejected. The detailed step-by-step preprocessing scripts and parameters are detailed in Appendix – A.

The artifact-free data were segmented into 4-second epochs, 2 s before and 2 s after each trigger (Toro et al. 1994). Trials with discontinuity and with an RT above ± 2 standard deviations from each participant’s mean were excluded (Berger and Kiefer 2021). After exclusion of artefacts and outlier trials, an average of 53 (45–58) trials for the DIR condition and 54 (51–57) trials for the MIR condition were used to extract ERP and ERO respectively.

### Analysis of the Event-Related Potentials (ERP) and Oscillations (ERO)

Due to varying RT across trials, EEG data were segmented using two separate triggers. First, the epoching was triggered by the time point when the participant had to initiate the reaching movement to target as the target object’s color turned blue, while another epoching was triggered by the time point as the participant’s index finger reached the target, which turned the target object’s color turned green. The dual-trigger approach effectively decoupled the neural signatures from the variance in total reaching duration. Using these two sets of segmented data we could analyze the ERPs and EROs related to the following two periods:

#### Stimulus-cued Initiation of Reaching (sReach)

The period starts when the target object turns blue, and participants start moving their non-dominant (in this case, left) hand’s index finger toward it, lasting for variable times among trials and participants of approximately 600 ms.

#### Finish of Reaching (fReach)

When participants reached the target object with their non-dominant hand’s index finger, the target object’s color turned green. The ERPs and EROs around this time point were analyzed to study the neural activities associated with the approximation to the target and the sensory feedback produced by the appearance of the green color.

A 30 Hz low-pass filter was applied to the continuous EEG data before ERP analysis. This filter was utilized to weaken task-irrelevant high-frequency noise and myogenic artifacts, thereby improving the signal-to-noise ratio and facilitating the visualization and quantification of slow cortical potentials (e.g., P3 and RRN). For ERP analyses, two separate baseline correction intervals were selected, one for the sReach and one for the fReach analyses. For sReach analyses the 100 ms before the stimulus that initiates the reaching movement was used as the baseline period, while for the fReach analyses the time period between − 1100 to -1000 ms relative to the reaching time point was used as the baseline period in order to be sure that this period precedes the initiation of the movement in most (80%) of all trials. To ensure baseline neutrality, a non-parametric cluster-based permutation test was performed on the raw EEG data within the baseline windows (− 1100 to − 1000 ms). No significant differences were found between conditions (t_sum_ = 752.62, *p* = 0.08), confirming that the choice of baseline did not introduce systematic bias into the subsequent ERP analyses.

For the characterization of the EROs in the above mentioned segments of the data, the total activity including both phase-locked and non-phase-locked EEG oscillations to the events were analyzed by computing complex Morlet wavelet transform (WT) with 0.5 Hz frequency resolution on single trials and ensemble averaging the magnitudes across the trials (Basar et al. 2001; Demiralp and Ademoglu 2001; Herrmann et al. 2014). The mean amplitudes of the following frequency bands were analyzed: theta band (4–8 Hz), alpha band (8–13 Hz), and beta band (13–30 Hz). For the 0.5–15 Hz frequency range 3-cycle wavelets and for the 15–45 Hz frequency range 7-cycle wavelets were used. Since each wavelet function has a certain temporal width, the baseline correction periods were kept longer for representing at least few cycles of an oscillatory activity. They were also set to earlier time regions to avoid any spread of activity between the baseline periods and the analyzed time ranges. Therefore, for the sReach period the baseline was set between the − 500 and − 100 ms before the stimulus that initiates the reaching movement and for the fReach period between the − 1500 and − 1100 ms before the time point of reaching the target. To ensure baseline neutrality, a non-parametric cluster-based permutation test was performed on the raw EEG data within the baseline windows (− 1500 to − 1100 ms). No significant differences were found between conditions in delta (t_sum_ = -52.87, *p* = 0.31), theta (t_sum_ = 32.95, *p* = 0.40), alpha (t_sum_ = 87.17, *p* = 0.18), beta (t_sum_ = 34.57, *p* = 0.33) and gamma (t_sum_ = -80.53, *p* = 0.13) confirming that the choice of baseline did not introduce systematic bias into the subsequent ERO analyses.

### Statistical Analysis

The times to reach the target for DIR and MIR conditions were analyzed using IBM SPSS (v. 29.0). The normality of the data distribution was assessed using the Shapiro-Wilk test (Shapiro and Wilk 1965), histogram graphs, and skewness-kurtosis values due to the sample size being less than 30 (King and Eckersley 2019). For parametric variables, paired sample t-tests were used, and for non-parametric variables, Wilcoxon Signed Rank tests were employed.

For statistical comparisons of EEG data, cluster-based non-parametric permutation tests with the maximum number of Monte Carlo simulations (2^15^ = 32768) were applied using the Fieldtrip (Oostenveld et al. 2011) software developed in MATLAB. While strictly controlling the Family-Wise Error Rate (FWER) over the spatial and temporal dimensions, this method inherently resolves the multiple comparisons problem across continuous EEG time points and electrode arrays by evaluating the data at the cluster level rather than the individual sample level. (Maris and Oostenveld 2007). For the Event-Related Oscillations (ERO), analyses were conducted independently within a priori defined frequency bands (e.g., theta, alpha, beta). To report the magnitude and spatiotemporal extent of the identified effects, the cluster-level test statistic (the sum of the t-values within the significant cluster, denoted as t_sum_) is reported alongside the exact permutation p-values.

## Results

### Reaching Time

The grand-mean of all participants’ reaching times were calculated as the mean of each subject’s individual median reaching time, yielding 605.77 ± 79.43 ms for the DIR condition and 650.52 ± 102.88 ms for the MIR condition. The Shapiro-Wilk test, histogram analysis and skewness-kurtosis values indicated that the reaching times were normally distributed for both conditions. So, paired t-test was conducted to evaluate statistically significant differences between the two conditions across all participants, showing that the time to reach the target was significantly longer in the MIR compared to the DIR condition [t (14) = -3.699, *p* = 0.006].

### Event-Related Potentials (ERPs)

#### ERPs Associated with the Stimulus-Cued Initiation of Reaching Movement (sReach)

**Fig. 1:**
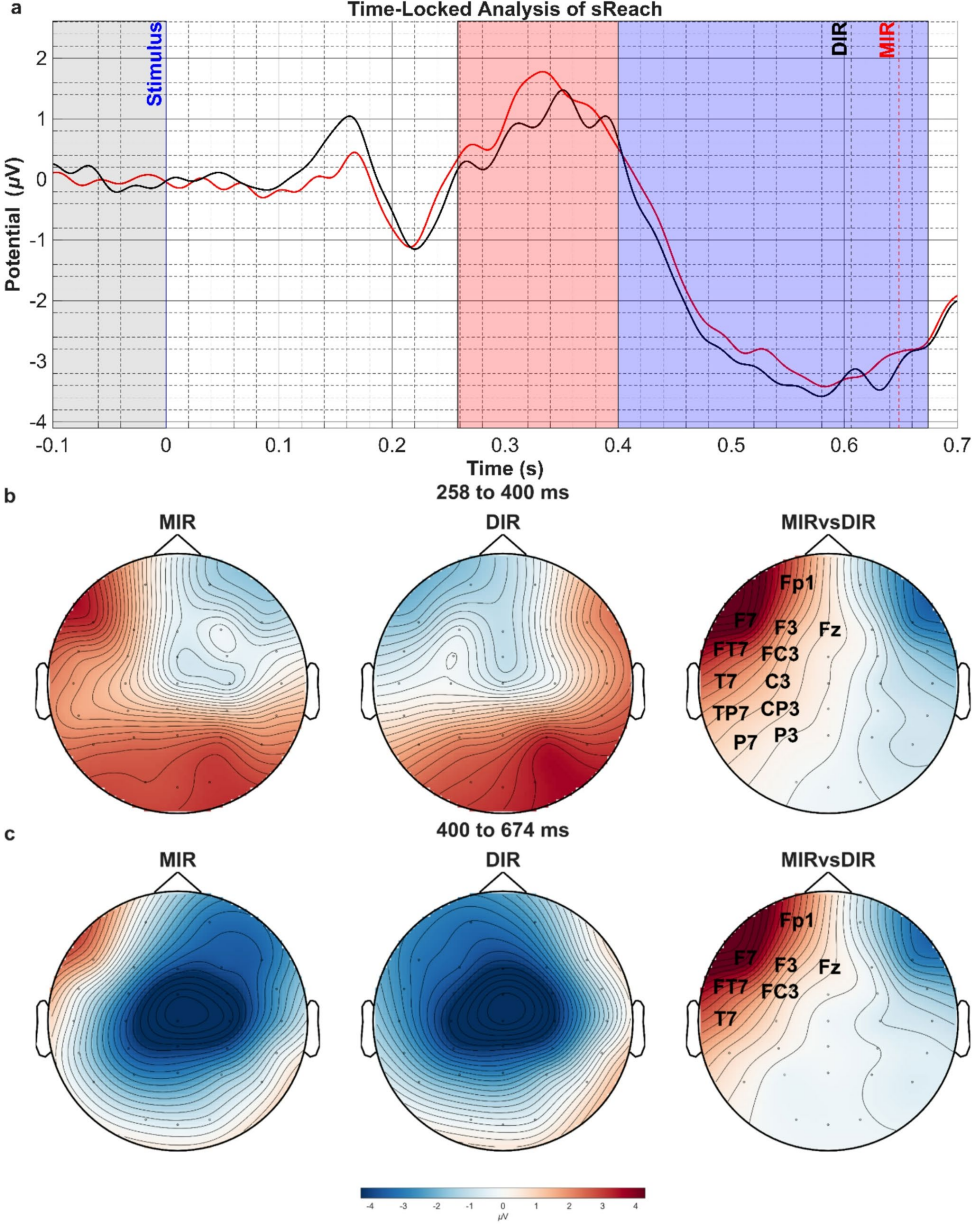
**a**) Comparison of the ERPs in MIR vs. DIR conditions in the sReach period. The traces correspond to the average voltages in all channels. Pale grey area corresponds to the baseline and significant voltage difference is found in pale red area (t_sum_=7807.04, *p* = 0.01). Vertical blue solid line corresponds to the visual cue that initiates the reaching trial when the target object’s color turns in blue (t = 0). Dashed black and red lines indicate grand-mean of reaching times for DIR and MIR conditions respectively. **b**) The topographic distribution of voltages at 258–400 ms in both conditions (left and middle topographies) and difference between the two conditions (right topography). **c**) The topographic distribution of voltages at 400–674 ms in both conditions (left and middle topographies) and difference between the two conditions (right topography). The channel names on the right topography represent the statistically significant spatial cluster (t_sum_=7807.04, *p* = 0.01)

Figure 1 shows the grand averages of EEG segments from all channels and participants, covering 100 ms before and 700 ms after the initiation of the reaching movement triggered by the target object turning into blue color (t = 0, marked by the blue vertical line). The grand-mean of reaching times to the target are indicated by the dashed vertical black and red lines for DIR and MIR, respectively. In both conditions, a sequence of waves was observed up to 400 ms after the stimulus that initiates the reaching movement, followed by a steep negative shift, that reaches its peak value shortly before reaching the target. Statistical comparison between the MIR and DIR conditions revealed a significant cluster within the 258 to 674 ms time period with significantly more positive values for MIR compared to DIR condition (t_sum_=7807.04, *p* = 0.01, Fig. 1a). The time courses of the ERPs could be divided into a positive wave between 258 and 400 ms and a slow negative deflection between 400 and 674 ms. The positive wave resembling a P3 wave displayed a symmetric parietal distribution with an additional frontocentral positivity contralateral to the moving hand in the DIR and to the virtually moving hand in the MIR condition (Fig. 1b, left and middle topographies). The significant difference between the two conditions stemmed from a stronger left frontocentral positivity in the MIR compared to the DIR condition (Fig. 1b, right topography). The later negative wave displayed a midcentral maximum in both DIR and MIR conditions, which was accompanied by frontocentral positivities similar to those in the earlier time window (Fig. 1c, left and middle topographies). The difference between the two conditions was again due to a more positive potential in left frontocentral area in MIR compared with DIR condition (Fig. 1c, right topography).

#### ERPs Associated with the Finish of Reaching Movement (fReach)

**Fig. 2:**
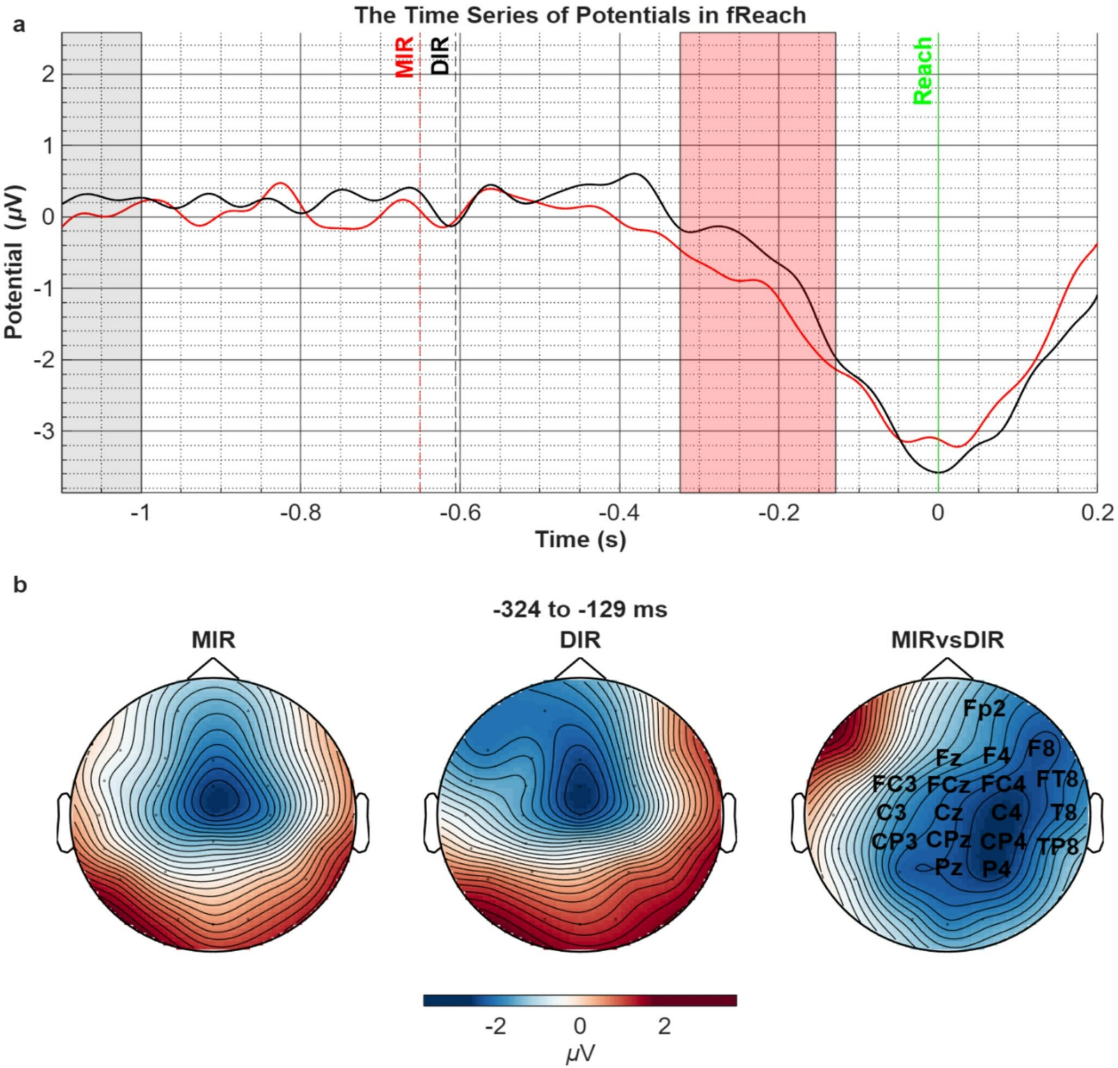
(**a**) Comparison of the ERPs in MIR vs. DIR conditions in the fReach period. The traces correspond to the average voltages in all channels. Pale grey area corresponds to the baseline and significant voltage difference is found in red shaded area (t_sum_=-5068.72, *p* = 0.009). Vertical green solid line corresponds to the finish of the movement when the target object’s color turns green (t = 0). Dashed black and red lines correspond to the approximated stimulus that initiate the reaching movement estimated by the grand-mean of reaching times for DIR and MIR conditions, respectively. (**b**) The topographic distribution of voltages in both conditions (left 2 topographies) in the time period that revealed significant difference between the two conditions (right topography). The channel names on the right topography represent the statistically significant spatial cluster (t_sum_=-5068.72, *p* = 0.009)

Due to the varying speeds among the individuals and trials, a separate segmentation and averaging process was performed to examine potential changes related to the moment of reaching the target. The grand averages of EEG segments, aligned to the moment of reaching the target (t = 0, solid vertical green line), calculated across all channels and participants, are shown in Fig. 2a. This time, the dashed black and red vertical lines represent the stimulus that initiates the movement that estimated using the grand-mean reaching times. The signals were baseline-corrected according to the mean voltages in the − 1100 to -1000 ms time interval, since this interval precedes the initiation time point of the movements for most of the trials (80%).

Before the movement finished, average potential of all channels displayed a negative shift with a midcentral peak in both the DIR and MIR conditions (Fig. 2a), which returned to the baseline after finishing the reaching movement. To evaluate the differences in this period preceding the finishing of the reaching movement, voltages in the − 700 to 0 ms according to the reaching time point were statistically compared between MIR and DIR conditions. The results revealed a significant difference between − 324 and − 129 ms (t_sum_=-5068.72, *p* = 0.009) for the MIR condition (Fig. 2b). This increased negative shift in the MIR condition was distributed along the frontoparietal channels with a slight shift to the right hemisphere.

### Event-Related Oscillations (ERO)

Significant power changes in the theta, alpha/mu and beta bands were observed in the EEG during the stimulus that initiation and finish of the reaching movement. These changes are detailed below.

#### EROs Associated with the Stimulus-Cued Initiation of Reaching Movement (sReach)

##### Alpha rhythm

**Fig. 3:**
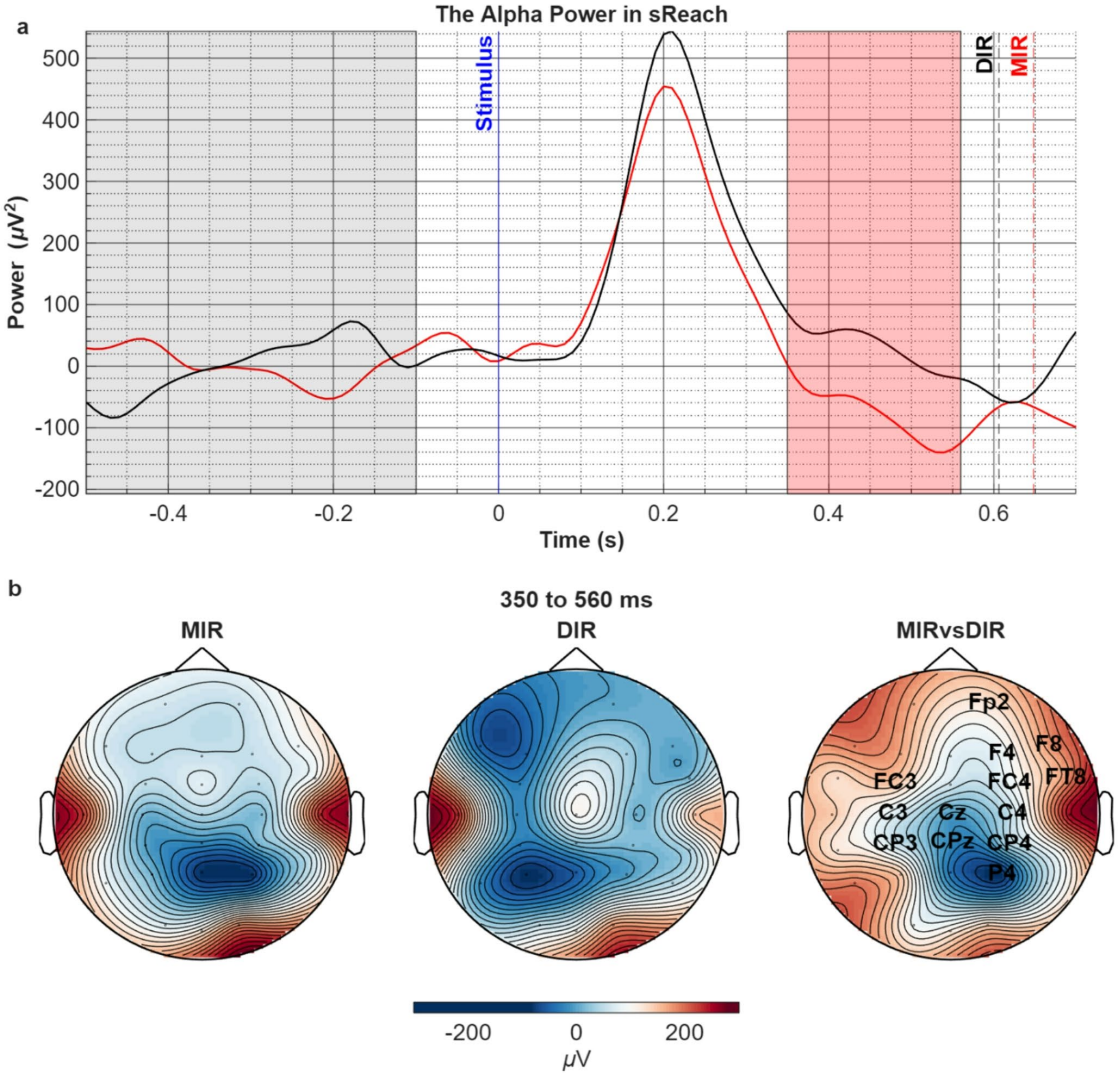
**a**) Comparison of the alpha power in MIR vs. DIR conditions in the sReach period. The traces correspond to the average power in all channels. Pale grey area corresponds to the baseline and significant voltage difference is found in the red shaded area (t_sum_=-464.60, *p* = 0.01). Vertical blue solid line corresponds to the stimulus-cued initiation of the movement when the target object’s color turns blue (t = 0). Dashed black and red lines indicate grand-mean of reaching times for DIR and MIR conditions respectively. **b**) The topographic distribution of alpha power in both conditions (left 2 topographies) in the time period that revealed significant difference between the two conditions (right topography). The channel names on the right topography represent the statistically significant spatial cluster (t_sum_=-464.60, *p* = 0.01)

After the target object’s color turns blue, which initiates the reaching movement, a notable alpha (8–13 Hz) power increase was observed in both conditions, peaking at approximately 200 ms, with no statistically significant difference between two conditions. However, following this period, a significant difference was observed between the two conditions in the 350–560 ms interval (Fig. 3a). The topographical distribution (Fig. 3b) showed that this difference stemmed from a stronger right centroparietal alpha ERD in the MIR compared with the DIR condition (t_sum_=-464.60, *p* = 0.01).

##### Beta rhythm

**Fig. 4:**
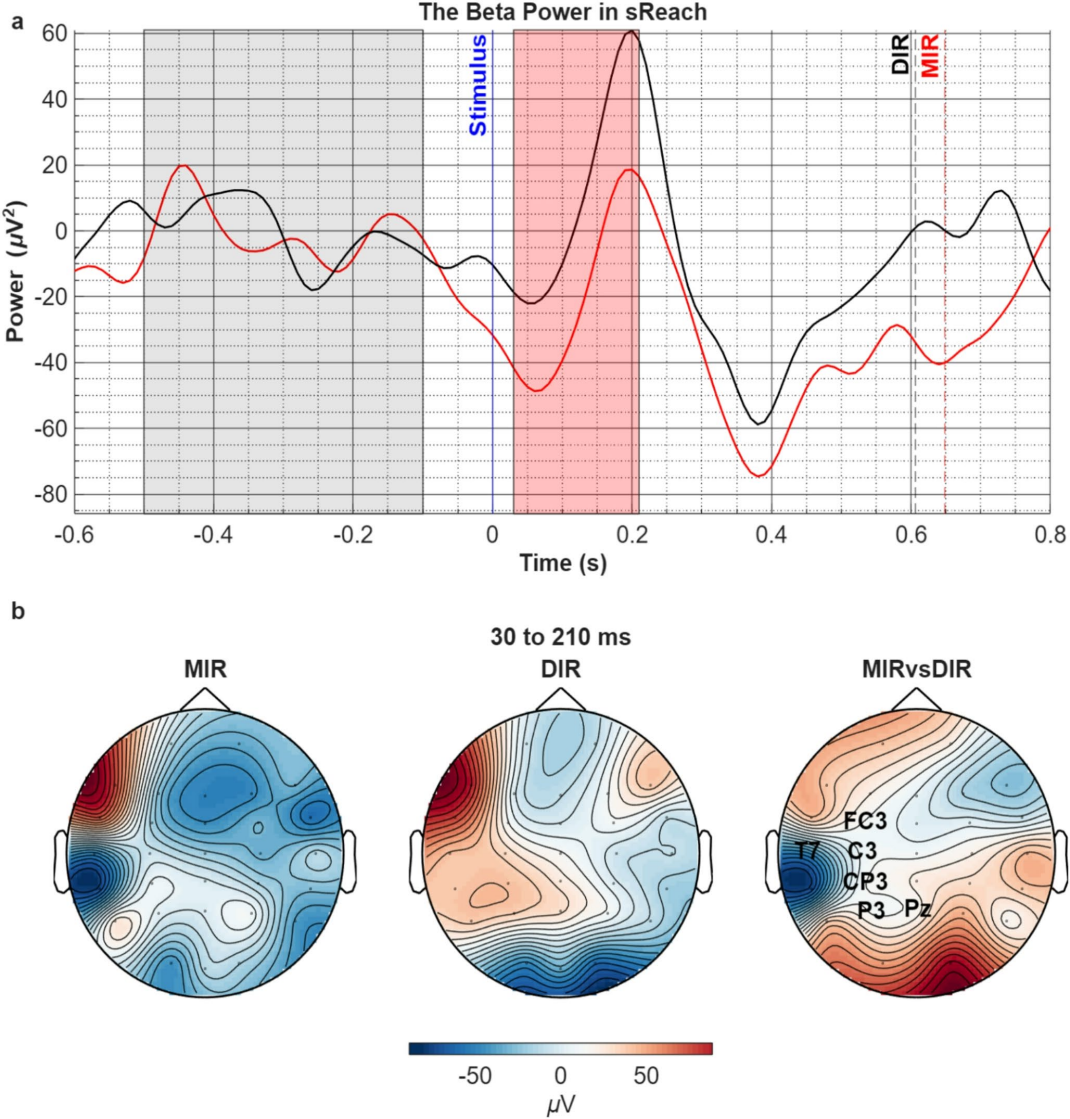
**a**) Comparison of the beta power in MIR vs. DIR conditions in the sReach period. Pale grey area corresponds to the baseline and significant voltage difference is found in pale red area (t_sum_=-302.14, *p* = 0.04). Vertical blue solid line corresponds to the stimulus-cued initiation time of the movement when the target object’s color turns blue (t = 0). Dashed black and red lines indicate grand-mean of reaching times for DIR and MIR conditions respectively. **b**) The topographic distribution of beta power in both conditions (left 2 topographies) in the time period that revealed significant difference between the conditions (right topography). The channel names on the right topography represent the statistically significant spatial cluster (t_sum_=-302.14, *p* = 0.04)

In both conditions, after the target object’s color turned blue to initiate the reaching movement, beta frequency band (13–30 Hz) displayed a synchronization peaking at around 200 ms similar to the synchronization observed in the alpha band (Fig. 4a). However, in contrast to alpha power which showed no difference between the two conditions during this time period, beta power exhibited a clear increase in both left frontoparietal and right frontal areas in the DIR condition, with a significant reduction of power in the MIR condition within the 30 ms to 210 ms interval (t_sum_=-302.14, *p* = 0.04). The topography of this relative beta desynchronization in the MIR condition showed a left precentral to postcentral distribution (Fig. 4b).

#### EROs Associated with the Finish of Reaching Movement (fReach)

Due to the varying durations of the reaching movement among individuals, time-frequency analyses were repeated on EEG segments time-locked to the moment of reaching the target. The baseline for these analyses was set between − 1500 and − 1100 ms, since this interval precedes the initiation time point of the movements for most of the trials (80%).

##### Theta rhythm

**Fig. 5:**
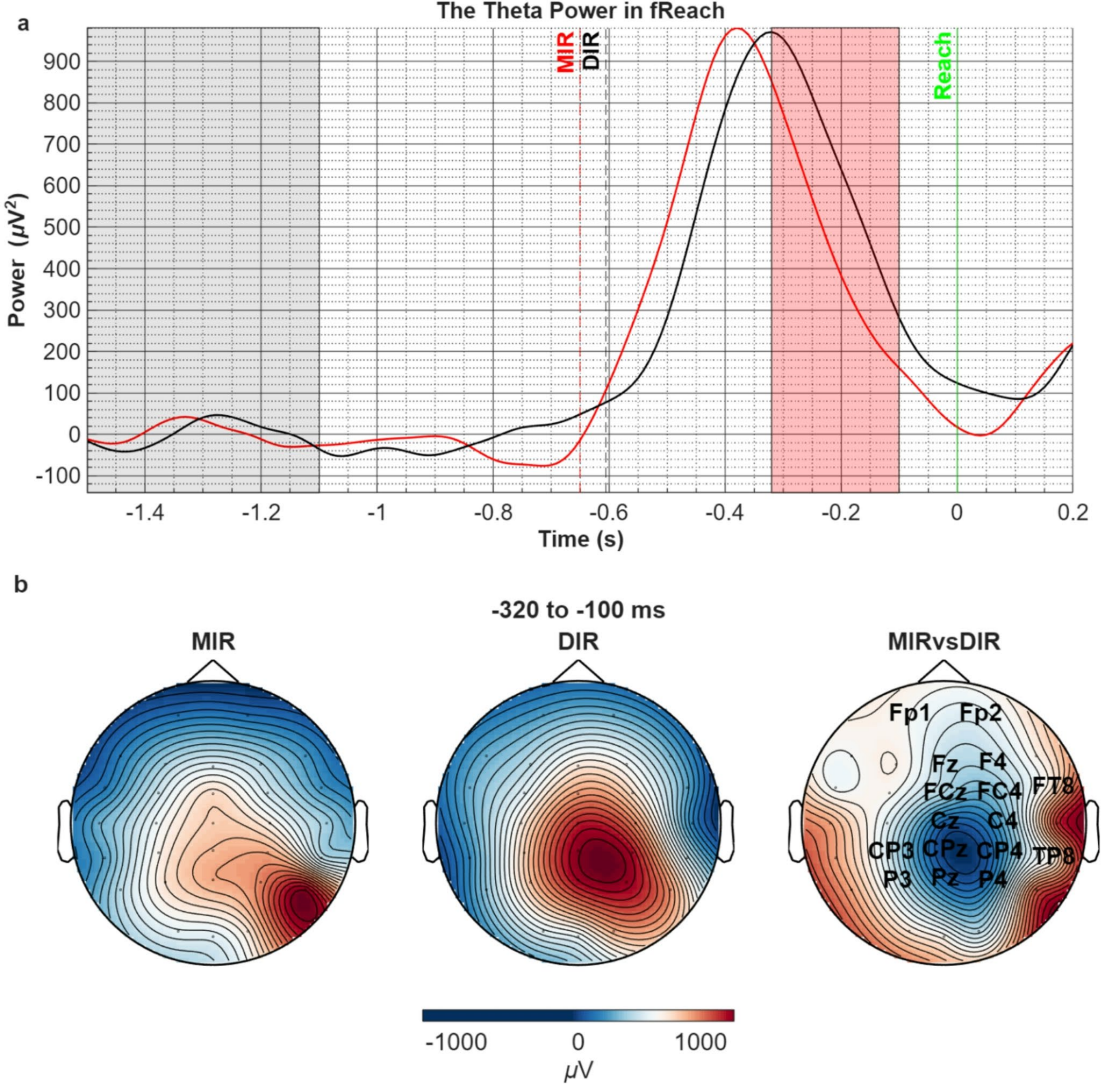
**a**) Comparison of the theta power in MIR vs. DIR conditions in the fReach period. Pale grey area corresponds to the baseline and significant power difference is shown with red shading (t_sum_=-847.09, *p* = 0.006). Vertical green solid line corresponds to the finish of the movement when the target object’s color turns green (t = 0). Dashed black and red lines correspond to the approximate the stimulus-cued initiation of the reaching movement estimated by the grand-mean of reaching times for DIR and MIR conditions, respectively. **b**) The topographic distribution of theta power in both conditions (left 2 topographies) in the time period that revealed significant difference between the conditions (right topography). The channel names on the right topography represent the statistically significant spatial cluster (t_sum_=-847.09, *p* = 0.006)

Changes in theta band power (4–8 Hz) before the completion of the reaching movement are shown in Fig. 5. The theta power, which initially showed a clear increase after the target appearance in both conditions, became desynchronized close to the reaching moment (t = 0, solid vertical green line) in significantly differing rates in the MIR and DIR conditions. This resulted in a statistically significant difference between the two conditions in the − 320 to -110 ms interval (t_sum_=-847.09, *p* = 0.006). As shown in Fig. 5b, this difference stems from an earlier starting theta desynchronization in the MIR compared with the DIR condition in bilateral centroparietal and right lateralized frontocentral areas.

##### Alpha Rhythm

**Fig. 6:**
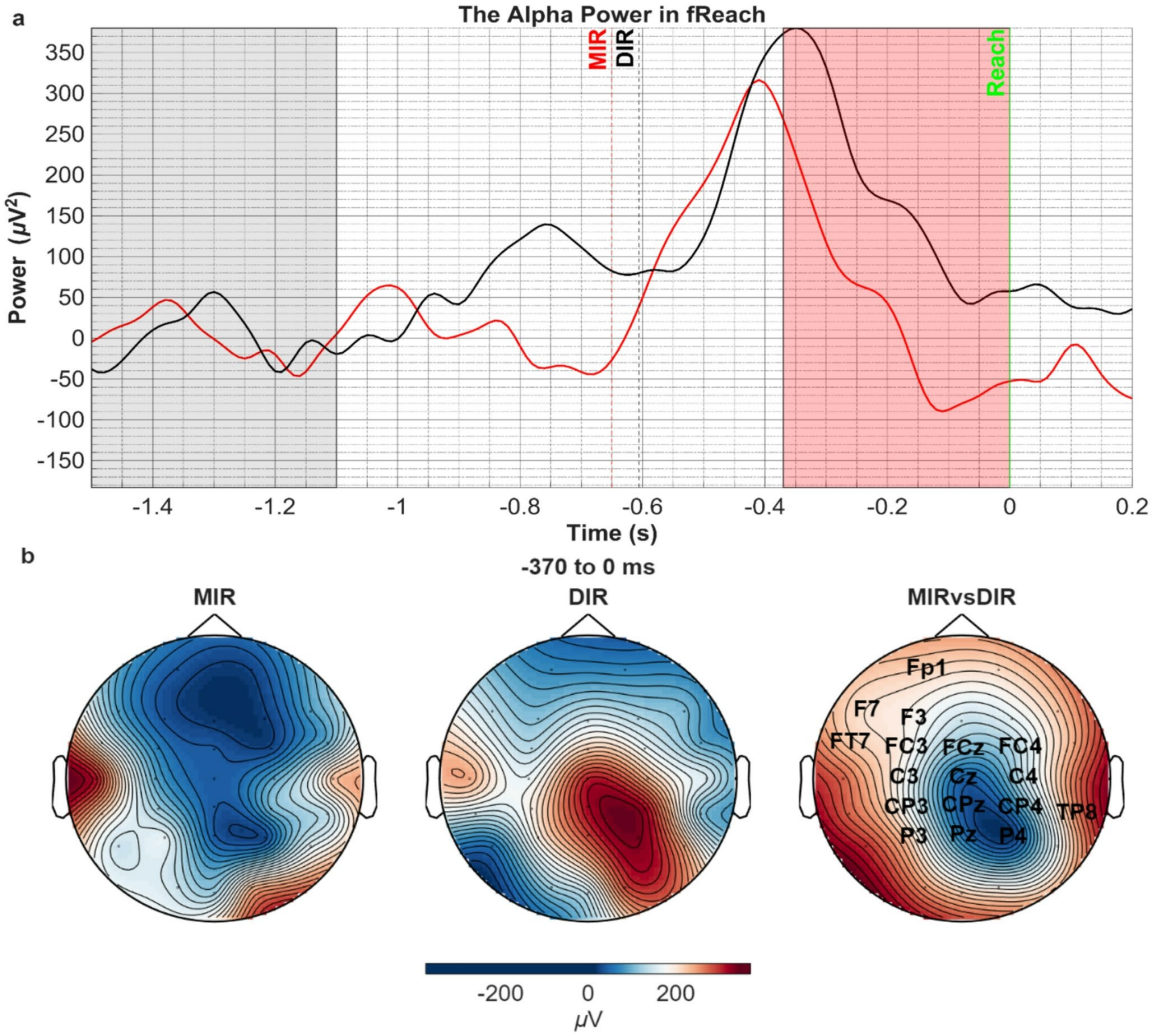
**a**) Comparison of the alpha/mu power in MIR vs. DIR conditions in the fReach period. Pale grey area corresponds to the baseline and significant voltage difference is found in pale red area (t_sum_=-833.28, *p* = 0.002). Vertical green solid line corresponds to the finish of the movement when the target object’s color turns green (t = 0). Dashed black and red lines correspond to the approximate stimulus-cued initiation time of the reaching movement estimated by the grand-mean of reaching times for DIR and MIR conditions, respectively. **b**) The topographic distribution of mu power in both conditions (left 2 topographies) in the time period that revealed significant difference between the conditions (right topography). The channel names on the right topography represent the statistically significant spatial cluster (t_sum_=-833.28, *p* = 0.002)

Preceding the reaching time, a statistically significant alpha power difference was obtained between the two conditions in the − 370 to 0 ms interval (t_sum_=-833.28, *p* = 0.002) (Fig. 6). The alpha power time series aligned to the finishing time-point of the reaching movement showed that power first increased in both conditions after the target appeared that initiated the reaching movement. Before the finish of the reaching movement, this alpha ERS became desynchronized, which, however, occurred earlier in the MIR compared with the DIR condition, leading to the significant alpha power difference (Fig. 6a). The topographic representation of the stronger alpha ERD in the MIR condition showed a bilateral centroparietal distribution.

## Discussion

In line with our first hypothesis, EEG responses during a center-out reaching task with visual feedback based on the avatars of the moving and mirrored hand in an immersive VR environment showed evident differences that can be interpreted within the framework of predictive coding theory. Although the reaching times were different between the DIR and MIR conditions, the use of dual time-locking to the stimulus-locked initiation (sReach) and end (fReach) of reaching movement ensured that electrophysiological differences could be attributed to distinct processing phases rather than being affected by different movement durations. An inherent challenge in locking epochs to the terminal phase of variable-duration movements is baseline selection. Because reaching times were significantly longer in the MIR condition, any potential baseline overlap with early motor negativities would disproportionately shift the MIR waveforms in a positive direction. Consequently, the significantly larger RRN found in the MIR condition represents a conservative estimate, confirming the robustness of the condition contrast despite the chronometric variability.

The results demonstrated the emergence of robust behavioral, ERP (Event-Related Potentials) and ERO (Event-Related Oscillations) patterns, pointing to three key findings: (1) reaching movements under mirrored visual feedback were behaviorally more effortful, as reflected by longer movement completion times; (2) ERP analyses revealed an enhanced P3 component aligned to the stimulus that initiated the movement and a distinct slow negative shift aligned to the reaching time point, which we called reaching-related negativity (RRN); (3) time–frequency analyses identified stronger alpha-ERD and reduced beta-ERS aligned with the start and earlier starting theta and mu-ERDs aligned with the reaching time point under mirrored feedback condition. The topographies of these oscillatory signals pointed either to motor areas that could produce internal models for the control of the mirrored hand or to brain areas that are engaged in directing spatial attention. Together, these findings may indicate that mirrored visual feedback modulates both cognitive and motor cortical processes associated with planning and visuomotor adaptation of the forward models of the mirrored hand movement. The key findings of this study are summarized in Table 1.

**Table 1:** Summary of EEG Findings and Their Proposed Physiological Interpretations

EEG Component (Time Window)	Topography	Difference	Proposed Mechanism/Interpretation
P3/P300 (sReach) 258–400 ms	Left frontocentral	↑ Positivity in MIR (*p* = 0.01)	**Increased Attentional and Stimulus-Response Coupling Demand** Consistent with resource allocation models (Kok 2001), the amplitude increase reflects higher cognitive demand required to process incongruent stimulus-response (S-R) couplings in the non-dominant hand (Kida et al. 2023).
Beta-ERD (sReach) 30–210 ms	Left centroparietal	↓ Power in MIR (*p* = 0.04)	**Early Motor System Engagement** Indexes early activation of the motor networks corresponding to the visually mirrored hand. Consistent with classical baseline ERD models where cortical networks un-idle to prepare for complex motor execution (Chatrian et al. 1959; Neuper and Pfurtscheller 2001).
Alpha-ERD (sReach) 350–560 ms	Right centroparietal	↑ Power in MIR (*p* = 0.01)	**Mirror Neuron System (MNS) Activation** Matches established patterns of Mu-rhythm suppression during action observation (Muthukumaraswamy et al. 2004), reflecting the engagement of premotor-parietal MNS networks processing the visual avatar (Arnstein et al. 2011).
RRN (fReach) –324 to − 129 ms	Frontoparietal, right lateralized	↑ Negativity in MIR (*p* = 0.009)	**Online Sensorimotor Integration** Represents continuous neural effort required for movement monitoring and resolving incongruent visual-proprioceptive feedback. This acute cortical engagement may serve as a driver for the long-term neuroplasticity seen in clinical mirror therapy (Michielsen et al. 2011).
Theta-ERD (fReach) –320 to − 110 ms	Bilateral centroparietal + right frontocentral	Earlier ERD in MIR (*p* = 0.006)	**Cognitive Control and Feedback Monitoring** Reflects the top-down cognitive control (Cavanagh and Frank 2014) deployed to override automatic motor habits and continuously evaluate visual feedback during a complex, visually guided reach (Borra et al. 2023; Chung et al. 2017).
Alpha-ERD (fReach) –370 to 0 ms	Bilateral frontocentroparietal	Stronger ERD in MIR (*p* = 0.002)	**Sustained MNS Engagement** Indicates that the specific motor networks activated by observing the mirrored hand (Muthukumaraswamy and Johnson 2004) remain actively engaged right up until the exact moment of target acquisition.

RRN = Reaching-related Negativity; ERD = Event-Related Desynchronization; ERS = Event-Related Synchronization

### Reaching-Related Neural Processing

A key contribution of this study is the identification of the RRN, a negative-going ERP component that emerges shortly before movement completion. By additionally aligning EEG epochs to the exact reaching moment—rather than only to the start of the movement—we revealed signal features that remain hidden in traditional stimulus-locked analyses. In both the DIR and MIR conditions, we observed a pronounced negative shift with a mid-frontocentral maximum beginning roughly 400 ms before and peaking precisely at the time of reaching. While the RRN likely shares underlying neural substrates with broader performance monitoring systems, its specific temporal locking to the terminal phase of a continuous reach in the VR environment identifies it as a distinct mechanism for the high-level integration of visual and proprioceptive feedback during the resolution of visuomotor conflict. This component, centered over the supplementary motor area, appears distinct from classical motor-related potentials such as the readiness potential (Bereitschaftspotential) or contingent negative variation (CNV), which typically precede and prepare discrete motor onsets like button presses; while movement-related cortical potentials like the Bereitschaftspotential and CNV are onset-locked and reflect preparation (Deecke 1996; Kornhuber and Deecke 2016; Shibasaki and Hallett 2006; Walter et al. 1964), the RRN is completion-locked and reflects the terminal phase of the reaching trajectory. Furthermore, the RRN differs from feedback-related negativity (FRN); whereas the FRN is a post-hoc response to a discrete outcome (Kirsch et al. 2022; Nieuwenhuis et al. 2005), the RRN is an anticipatory negative shift that increases as the hand approaches the target. The RRN likely reflects ongoing monitoring and sensorimotor adjustment during the final phase of movement, consistent with predictive coding accounts. This interpretation frames motor control as a continuous predictive feedback process rather than a purely feedforward command structure (Shadmehr and Krakauer 2008). The stronger RRN observed in the mirrored condition seems to reflect the additional computational demand of verifying whether the visually transformed limb has reached the intended endpoint. We interpret this as evidence that mirrored feedback not only alters movement kinematics but also specifically engages neural mechanisms that reconcile predicted and actual sensory outcomes during goal verification. This suggests that the RRN, which seemingly represents the computational effort of integrating continuous, and in our case, incongruent, sensory-motor feedback during the movement itself, differs from similar negative potential shifts already described in the literature. To our knowledge, this ERP pattern time-locked to movement completion has not been previously described and may help refine theoretical models that attribute late-stage movement activity to feedback monitoring and performance evaluation.

### Motor System Engagement and Visuomotor Adaptation

After the target location was presented and the reaching movement was initiated, a positive ERP peak emerged at roughly 300 ms. Because the target direction should be clearly identified by this time, this ~ 300 ms positivity corresponds well to a P3 component, which is typically linked to decision regarding the target position. Beyond the expected bilateral parietal distribution of the P3, the topography also showed frontocentral positive deflections—contralateral to the moving hand in the DIR condition and contralateral to the mirrored hand in the MIR condition. This pattern may indicate that the frontocentral activity reflects visual feedback used to update the internal forward model. The key difference between the two conditions was a stronger positivity in the left frontocentral region in the MIR condition, contralateral to the virtually moving hand. This enhanced activity probably represents cortical updating of the internal forward model in response to prediction error caused by the incongruent visual reafference in the MIR condition (Jeannerod 2003; Kilner et al. 2007). This result also supports the idea that viewing the right-hand avatar engages related motor areas through visual feedback (Rizzolatti and Sinigaglia 2016). In addition, in line with the theoretical framework proposed by (Kok 2001),the significantly larger P3 amplitude observed in the MIR condition may reflects a higher allocation of processing capacity and attentional resources required to evaluate the cognitively incongruent mirrored feedback. This is specifically relevant to our mirrored paradigm, where the brain must process an incongruent visual-motor mapping. As demonstrated by (Kida et al. 2004, 2012, 2023), this modulation reflects the effort involved in maintaining stimulus-response (S-R) coupling when task demands are complex. Given our use of the non-dominant hand, the MIR condition likely represents a significant S-R decoupling, necessitating higher-level monitoring and recruitment of neural resources, as evidenced by both the P3 enhancement.

The beta oscillations further support the motor system involvement during the mid-sagittal mirroring condition. A significant difference in beta power between the MIR and DIR conditions emerged within 30 to 210 ms after movement onset on the left central area. Typically, beta event-related synchronization (ERS) is linked to the maintenance of a stable motor state or movement inhibition, whereas beta event-related desynchronization (ERD) indicates active motor engagement and sensorimotor processing (Engel and Fries 2010). In the DIR condition, a pronounced beta ERS appeared over the left frontocentral region, pointing to the inhibition of the motor region ipsilateral to the moving hand. In contrast, beta power was markedly reduced in the left central area in the MIR condition, pointing to the superimposition of a beta ERD on the motor areas controlling the virtually mirrored hand due to the visual feedback.

The alpha and beta oscillations observed over the centroparietal electrodes also provides neurophysiological evidence for the recruitment of cortical areas corresponding to the mirrored limb. This is consistent with findings of mu-rhythm modulation during mirror-box tasks (Frenkel-Toledo et al. 2014; Muthukumaraswamy and Johnson 2004). Specifically, research combining EEG and fMRI (Arnstein et al. 2011) suggests that these oscillations may reflect activity within the inferior parietal lobule (IPL) and dorsal premotor cortex (PMd). As components of the mirror neuron system, these regions are likely involved in resolving the incongruence between actual proprioception and mirrored visual feedback. Consequently, the observed ERP and ERO modulations may serve as objective markers of the cortical engagement necessary for the mechanisms of recovery discussed in foundational MVF research (Ramachandran and Altschuler 2009).

Taken together, the ERP and oscillatory patterns point toward a shift in control strategy: under direct feedback, reaching relies more strongly on efficient forward models, whereas under mirrored feedback, the system engages in a more feedback-dependent mode requiring continuous recalibration of visual reafference and the efferent copy. Visual reafference probably generates indirect activations in and around motor areas that normally would control the movement of the mirrored hand avatar.

### Management of Attentional Resources

Compared to direct feedback, mirrored reaching was characterized by enhanced alpha desynchronization over parietal and central regions both in the analyses time-locked to the stimulus-cued initiation and to the finish of the reaching movement. These findings probably index increased visuospatial attention allocation. Alpha power, which initially peaked around shortly before the P3 peak after the appearance of the target position in both conditions, was followed by a significantly stronger alpha ERD, which was mostly expressed in the right centroparietal (Cz, C4, CP4, P4) areas. The enhanced alpha ERD particularly over right frontoparietal area suggests increased attentional deployment to resolve the mismatch between the visual reafference and the efference copy in the MIR condition (Thut et al. 2006).

Similarly, theta power also peaked in both conditions around the P3 peak after the visual presentation of the target position, and displayed condition-specific desynchronization afterwards. As the hand approached the target, theta power desynchronized earlier in the MIR condition, resulting in significantly lower power in the bilateral centroparietal and right frontocentral regions before the reaching time point. Among other correlations, theta oscillations have been associated with attention and feedback prediction (Basar et al. 1999; Cavanagh and Frank 2014; Demiralp and Ademoglu 2001). The earlier starting and stronger desynchronization of the theta activity in MIR compared with the DIR condition with right lateralization seems to reflect the stronger activation of the visuo-spatial attention system due to the discrepancy between the visual reafference and the efferent copy.

### Implications for VR-Based Mirror Therapy in Neurorehabilitation

Although this study was conducted in healthy participants, effects were obtained in a controlled, distraction-reduced VR setting, may indicating that VR can reproducibly evoke neural processes thought to underpin mirror-based mechanisms presumed in mirror therapy (MT) approach applied in persons with hemiplegia/hemiparesis. The findings have meaningful implications for mirror therapy and VR-based rehabilitation. The enhanced engagement of attention and motor regions under mirrored conditions suggests that VR-mediated mid-sagittal mirroring successfully recruits networks involved in spatial attention and contralateral motor control. This mechanism provides a theoretical basis for how such interventions might eventually be utilized to target and activate impaired networks in clinical populations, though this remains to be empirically tested in patients. While our findings provide a normative baseline regarding how the intact brain resolves visuomotor conflict during mirrored feedback, future clinical trials involving patients with specific motor deficits are required to determine whether these exact ERP and ERO signatures translate to actual motor recovery in damaged neural networks, any direct clinical implications must be interpreted with strict caution.

Considering the restricted task diversity and visual constraints of conventional mirror therapy, our neurophysiological data suggests that immersive VR could serve as a viable and engaging alternative platform for developing future rehabilitation protocols. Moreover, once validated in patient populations, neural metrics such as the RRN, theta, mu, and beta, have the potential to serve as objective metrics for monitoring cortical engagement or customizing therapy intensity. Evaluation of these EEG-based metrics on patients before or along MT may also help to develop closed-loop, personalized VR protocols where feedback difficulty or movement guidance adapts dynamically based on real-time neural state in the future.

## Conclusions

This study demonstrates that mirroring a movement within an immersive VR environment elicits reliable, mechanism-informative neural signatures measurable with EEG. We observed a range of other ERP and ERO components associated with both the initiating stimulus and reaching time point, that topographically can be associated either with increased spatial attention or activation of the motor areas responsible for the control of the mirrored hand.

Our analyses time-locked to the reaching time point uncovered signal features that, due to the varying reaching times across the trials, are hidden in the analyses time-locked to the stimulus that initiates the movement (Borra et al. 2023; Garipelli et al. 2021). The reaching-time-locked analyses revealed a clear frontocentral negative shift, the reaching related negativity (RRN), that starts before and peaks at the reaching time point and revealed significant change due to virtual mirroring of the limb movement.

The observed ERP and ERO differences between mirrored and direct conditions can be interpreted within a predictive coding framework of motor control, pointing toward a shift in motor control strategy from efficient forward models to a more feedback-dependent mode in the mirroring condition. It is important to note that because our experimental design utilized binary visual manipulation rather than a parametric gradation of sensory conflict, our findings cannot map the proportional scaling of prediction error. Therefore, while our data strongly aligns with an active inference framework, future studies employing graded visual perturbations are necessary to definitively isolate these predictive coding mechanisms.

While the sample size and electrode density were limited by the technical constraints of integrating EEG with immersive VR hardware, the use of cluster-based non-parametric permutation testing and high trial counts per condition ensured sufficient statistical power for within-subject comparisons. Future studies employing higher-density EEG or source-level modeling could further refine the spatial localization of the observed effects.

The recruitment of only male, while serving as a necessary methodological control for this proof-of-concept phase, limits the generalizability of the results across sexes. Future research should investigate the role of sex as a biological variable to determine if hormonal fluctuations influence the cortical processing of the virtual mirror illusion.

## Data Availability

The datasets used and/or analyzed during the current study are available from the corresponding author on reasonable request.

## Appendix – A: Steps for Artifact Elimination

Steps for artifact elimination are detailed below:

1. The dataset was loaded into the EEGLAB toolbox.

2. Channel location information was added to the dataset.

3. Portions of the dataset related to pre-experiment, practice trials, and post-experiment were removed.

4. To remove eye artifacts using Independent Component Analysis (ICA), the following steps were performed sequentially:

o A clone of the original data was created.

o A band-pass filter (1–30 Hz) was applied to clone data to minimize the effects of slow potential drifts (< 0.5 Hz) and muscle and power line noise (50 Hz).

o Time segments without any sequential triggers for 2500 ms after a trigger were deleted from the clone using customized scripts from ERPLAB software.

o The sampling rate of the clone was reduced to 100 Hz to speed up the ICA decomposition process.

o Overvoltage values were semi-automatically scanned in the temporary dataset using a sliding window of 200 ms with 50 ms steps (time windows were excluded if the peak-to-peak voltage exceeded 200 µV).

o The clone was decomposed into independent components using the extended Infomax algorithm (Lee et al. 1999).

o The independent components obtained from the clone were inspected topographically and spectrally to identify components representing eye artifacts.

o The spatial weight coefficients (topographies) of the independent components from the clone were transferred to the original data.

5. A band-pass filter (0.1–45 Hz) was applied to the original data, and the identified ICA components representing eye movements were removed.

6. Overvoltage values were semi-automatically identified in the original data using a sliding window of 200 ms with 50 ms steps (time windows were excluded if the peak-to-peak voltage exceeded 200 µV).

7. The dataset was visually inspected to identify artifacts not removed by automatic algorithms.

8. The cleaned data was saved for further data processing steps.

Statements and Declarations.

### Competing Interests

None of the authors have potential conflicts of interest to be disclosed.
